# Validity of self-reported height and weight for estimating prevalence of overweight among Estonian adolescents: the Health Behaviour in School-aged Children study

**DOI:** 10.1186/s13104-015-1587-9

**Published:** 2015-10-26

**Authors:** Katrin Aasvee, Mette Rasmussen, Colette Kelly, Elvira Kurvinen, Mariano Vincenzo Giacchi, Namanjeet Ahluwalia

**Affiliations:** Department of Chronic Diseases, National Institute for Health Development, 42 Hiiu Street, 11619 Tallinn, Estonia; National Institute of Public Health, University of Southern Denmark, Øster Farimagsgade 5A, Copenhagen K, 1353 Denmark; Health Promotion Research Centre, National University of Ireland, 12 Distillery Road, Galway, Ireland; United Laboratories, Department of Genetics, Tartu University Hospital, 6 Hariduse Street, 10119 Tallinn, Estonia; Department Molecular and Developmental Medicine, Centre of Research for Health Education and Promotion, University of Siena, Via A. Moro 2, 53100 Siena, Italy; Health Scientist, Hyattsville, Maryland USA

**Keywords:** Height/weight, Body mass index, Self-reports, Validity, Overweight, Puberty

## Abstract

**Background:**

Low to moderate agreement between self-reported and directly measured anthropometry is shown in studies for adults and children. However, this issue needs further evaluation during puberty, a period marked by several transitions. We examined the correspondence of BMI status based on self-reported versus measured anthropometric data among Estonian adolescents with a specific focus on gender and age differences.

**Methods:**

Self-reported height and weight were determined in a national representative sample of Estonian schoolchildren collected within the framework of the HBSC (health behaviour of school-aged children) survey. Self-reported and directly measured height and weight were collected from 3379 students (1071 aged 11, 1133 aged 13 and 1175 aged 15 years). The standardized HBSC questionnaire was used for collecting self-reported data; direct anthropometric measures were taken after the HBSC questionnaires were completed. The accuracy of the self-reported values by age and gender groups were determined by comparing mean differences, Bland–Altman plots with limits of agreement, Kappa statistics, and by estimation of the sensitivity and positive predictive value for detecting overweight.

**Results:**

Mean self-reported weight, height and body mass index (BMI) values were significantly lower than corresponding values obtained using direct measurements. Mean differences between self-reported and directly measured weight, height and BMI were largest among 11-year-olds and smallest among students aged 15 years. Underestimation of overweight prevalence (includes obese) showed a graded trend which decreased in older age groups; the difference was greater among girls than boys in all age groups. The mean underestimation of overweight prevalence based on self-reported anthropometry was 3.6 percentage points. The positive predictive value was 72.3 % for boys and 63.4 % for girls.

**Conclusion:**

A distinct age-related pattern in underestimation of weight, height and prevalence of overweight was found; the bias decreased with increasing age. The mean underestimation of overweight prevalence based on self-reports was small, 3.6 %. Self-reported height and weight remain the method of choice in large surveys for practical and logistical reasons.

**Electronic supplementary material:**

The online version of this article (doi:10.1186/s13104-015-1587-9) contains supplementary material, which is available to authorized users.

## Background

Overweight and obesity in childhood continue to be public health concerns due to high prevalence, short and long-term adverse health consequences and increased health-care-costs [1–3]. Overweight and obesity are usually defined based on the body mass index (BMI) in relation to international reference standards [4, 5]. Although BMI is ideally based on actual measurement of height and weight, in large-scale epidemiological studies this is often not feasible. Thus self-reported values of height and weight are often used in national surveys in children [6] as well as in adults [7]. Validation studies comparing self-reported and direct measurements of height and weight indicate that both among adults and adolescents self-reports underestimate weight and BMI [8–15] while height values are often over-reported [11, 15–17]. Also, it is generally documented that the bias in self-reported weight and BMI is greater in females [13, 16, 18], in older people [13, 19] and in overweight subjects [8, 13, 20, 21]. The impact of age on validity of self-reported height and weight through adolescence is not clear because studies often report findings on a single age group [11, 21–24] or on a wide age-range [15, 20]. Two recent studies of adolescents show contradictory results on the influence of age on bias in self-reported weight [10, 18].

The 2009/2010 Health Behaviour in School-aged Children (HBSC) survey was conducted in nationally representative samples of 11-, 13-, and 15-year-old schoolchildren in more than 40 countries [25] using a standardized protocol and included self-reported data on age, height and weight. The age range sampled in the HBSC survey covers the period of rapid growth, and physical and emotional development [26]. Physical development in the years 11–15 is gender-specific, thus it is necessary to analyse the self-reported weight and height data by age-gender groups. To our knowledge, only two peer-reviewed validation studies on self-reported height and weight have been published on HBSC study samples. In a Welsh study of 15–17 year-old students a significant underestimation of weight and overweight prevalence (based on IOTF cut-offs; sample size was 418 students) was found: overweight prevalence was 6.4 percentage points lower when based on self-reported data. No gender differences were shown and the age-range was too narrow to evaluate differences by age [21]. In another validation study, based on a subset of Portuguese HBSC survey participants, the prevalence of overweight and obesity (based on IOTF cut-offs; sample size was 462 students) did not differ significantly when based on self-reports compared to direct measurements of height and weight [27]. However, the sample was too small to make conclusions regarding the effect of age on bias in BMI estimates.

In other adolescents’ surveys, the underestimation of overweight prevalence based on self-reported data compared to direct measures ranges from 2.6 % (18; overweight prevalence based on German age- and gender-specific cut-offs; sample size was 3468) to 15 % (23; overweight prevalence as determined based on IOTF cut-offs, sample size was 499 students] in European samples. Thus, overweight prevalence is quite different depending on use of self-reported or direct measurements to compute the BMI. In the Estonian HBSC 2005/2006 survey [6], in which BMI estimates were based on self-reports, the overweight prevalence among 15-year old girls was more than twofold lower than in the Countrywide Integrated Non-communicable Diseases Intervention (CINDI) monitoring which was based on direct measurements of height and weight (Unpublished observation by L. Suurorg, I. Tur; estimation of overweight was based on BMI ≥ 24 kg/m^2^). This finding motivated us to conduct the first of its kind, large-scale validation study among a nationally representative sample of Estonian youth (11–15 years old).

The overall aim of this study was to examine the agreement of BMI status based on self-reported versus measured anthropometric data in the 2009/2010 HBSC survey in Estonia. A specific focus of this paper was to evaluate age and gender based differences in the magnitude of bias in self-reported (versus measured) anthropometric data and respective prevalence of overweight.

## Methods

### Subjects

A nationally representative sample of 11-, 13- and 15-year-old Estonian schoolchildren was obtained as part of the WHO collaborative HBSC study in the academic year 2009/2010. Participants were selected using cluster sampling with school class as a sampling unit. Students completed the international standardized questionnaire in the classroom during one academic hour. Participation was voluntary and anonymous. The questionnaire and methodology for data collection and processing has been described elsewhere [6, 25]. The current analysis was performed on the Estonian national HBSC study sample which included 4914 subjects, with a response rate of 87.9 % from the primary sample of 5601 students. Non-participation was due to students being absent from school (n = 668), refusals (n = 10) or cases of incomplete or unreliable questionnaires (n = 9). After the cleaning process at the International HBSC databank (University of Bergen, Norway) the final Estonian national data set included 4224 subjects (2022 boys and 2205 girls). The standardized collection of questionnaire data was supplemented with direct measurements of students’ weight and height. In the final data set direct measurements of height and weight were available for 4171 students (99 %) and self-reported measures for 3424 students (81 %). Data with both self-reported and direct measurements of weight and height were used in the analysis for this paper. This sample included 3379 adolescents: 1071 aged 11 years (479 boys, 592 girls), 1133 aged 13 years (560 boys, 573 girls) and 1175 aged 15 years (555 boys, 620 girls).

### Anthropometric measures

The questions for recording weight and height were as follows: “How much do you weigh without clothes?” and “How tall are you without shoes?” Direct measurements of height and weight were taken using standardized portable equipment (Tanita HD365 for weighing and Tanita HR001 Leicester for height measures) and the guidelines of the CINDI Programme were followed [28]. The students were informed that their height and weight were to be measured after completing the HBSC survey. Two trained technicians took the measurements in the classroom when receiving the completed questionnaire from the student and registered values on the cover of the questionnaire. Majority (57 %) of the study technicians were healthcare professionals and remaining were university students. All technicians were provided detailed instruction on obtaining height and weight measurements using the same protocol. Height and weight were measured without shoes with a precision of 0.1 cm and 0.01 kg, respectively. Measurements were taken in the corner of the classroom, where only the student concerned was present. Adolescents were weighed with light indoor clothing and were asked to take off heavier accessories and remove personal items from their pockets. Standard weights for individual items of indoor clothing were collected earlier in a pilot study among 27 students. The list of weights of clothing in grams usually worn by students (see the Additional file 1) was used. The total weight of a student and weight of clothes were registered on the cover of the completed questionnaire from where the student weight without clothes could be estimated. Data collection was made by two groups of technicians: one was coordinated by National Institute for Health Development, Tallinn (10 technicians) and other by University of Tartu, Department of Public Health (11 technicians). After group training 5 sets of the portable Tanita equipment was provided to each group.

Body mass index (BMI) was calculated for self-reports and direct measures as weight in kg divided by the square of height in meters. Underweight, overweight and obesity were determined using the standardized age- and gender-specific IOTF (International Obesity Task Force) BMI cut-off points [4, 29]. In the present analysis the overweight category included both overweight and obese adolescents. The prevalence of obesity was too low (3.5 %) to conduct separate analyses by age and gender groups.

### Ethical approval

The survey was anonymous and voluntary, confidentiality of data was guaranteed. Parents and children were informed about the survey through the class-teachers before the study. School headmasters and representatives of parents gave written consent to participation prior to the survey day. Tallinn Medical Research Ethics Committee approved the questionnaire and research protocol (Application No. 901, Decision No. 1818, October 15th, 2009).

### Statistical analyses

As the validity of self-reported weight and height may differ by gender and age, separate analyses were conducted by gender and age group. SPSS version 15 and MedCalc version 12.4 were used for statistical analyses. Paired *T* test was used for comparisons of mean values, to evaluate associations between certain self-reported and measured values, Pearson’ correlation coefficients were calculated [30], and tests for one proportion were used to compare proportions [31]. Bland–Altman plots [32] were used to assess agreement between height and weight based on direct measurements (height-M, weight-M, respectively) and self-reported values (height-SR, weight-SR, respectively). Differences between measured and reported height and weight were plotted against the arithmetic mean of respective anthropometric values. Standard deviation (SD) of the differences was estimated and the 95 % limits of agreement (LOA) were calculated as the mean difference plus or minus 1.96 SD of the difference. The 95 % confidence intervals for upper and lower LOA were found.

Agreement of classification of underweight, normal weight and overweight based on measured and self-reported height and weight were assessed by weighted Kappa statistic [30]. Kappa values less than 0.20 are considered as “poor”, between 0.21 and 0.40 as “fair” agreement, between 0.41 and 0.60 as “moderate” agreement, between 0.61 and 0.80 as “good” agreement, and between 0.81 and 1.00 as “excellent” agreement. Analyses of sensitivity and specificity when using self-reports for estimation of overweight status were performed [33] and positive and negative predictive values were determined [34].

## Results

Comparisons of measured and self-reported weight, height and BMI with respective prevalence of overweight by gender and age are shown in Table 1. In almost all groups mean self-reported anthropometric variables were significantly lower than the measured values; the differences were not-significant for height data in 13- and 15-year-old girls and for BMI in boys aged 15.

**Table 1:** Characteristics of measured and self-reported weight, height, BMI and prevalence of overweight by gender and age groups of schoolchildren

	Weight (kg) mean ± SD	Height (cm) mean ± SD	BMI (kg/m^2^) mean ± SD	Overweight %
Total group (n = 3379)				
Measured value	54.37 ± 13.60	163.7 ± 10.85	20.1 ± 3.52	17.08
Self-reported value	52.94 ± 13.22*	163.1 ± 11.40*	19.7 ± 3.38*	13.50**
Mean difference	−1.44 ± 3.98	−0.5 ± 3.48	−0.4 ± 1.65	
Stratification by gender				
Boys (n = 1594)				
Measured value	56.55 ± 14.97	166.3 ± 12.41	20.2 ± 3.60	19.51
Self-reported value	55.21 ± 14.51*	165.6 ± 13.06*	19.9 ± 3.41*	16.38**
Mean difference	−1.34 ± 4.14	−0.8 ± 3.99	−0.3 ± 1.70	
Girls (n = 1785)				
Measured value	52.43 ± 11.92	161.3 ± 8.59	20.0 ± 3.44	14.85
Self-reported value	50.91 ± 11.57*	161.0 ± 9.16*	19.5 ± 3.35*	10.92**
Mean difference	−1.52 ± 3.83	−0.3 ± 2.94	−0.5 ± 1.60	
Stratification by age and gender				
Age 11, boys (n = 479)				
Measured value	45.20 ± 10.69	153.1 ± 7.57	19.1 ± 3.39	23.38
Self-reported value	43.32 ± 9.46*	152.2 ± 8.84*	18.6 ± 3.16*	19.00**
Mean difference	−1.88 ± 3.67	−0.9 ± 5.01	−0.5 ± 1.82	
Age 11, girls (n = 592)				
Measured value	44.61 ± 10.49	153.8 ± 7.27	18.7 ± 3.34	17.74
Self-reported value	42.51 ± 9.21*	152.9 ± 7.89*	18.1 ± 2.96*	12.33**
Mean difference	−2.10 ± 4.09	−1.0 ± 3.38	−0.6 ± 1.78	
Age 13, boys (n = 560)				
Measured value	56.55 ± 13.51	166.8 ± 8.85	20.2 ± 3.77	20.00
Self-reported value	54.96 ± 12.56*	165.8 ± 9.40*	19.9 ± 3.49*	16.43**
Mean difference	−1.59 ± 4.61	−1.0 ± 3.89	−0.4 ± 1.84	
Age 13, girls (n = 573)				
Measured value	54.05 ± 10.86	163.3 ± 6.34	20.2 ± 3.46	15.53
Self-reported value	52.45 ± 10.26*	163.0 ± 6.88	19.7 ± 3.45*	11.69**
Mean difference	−1.59 ± 4.26	−0.2 ± 3.11	−0.5 ± 1.87	
Age 15, boys (n = 555)				
Measured value	66.34 ± 12.41	177.3 ± 6.61	21.0 ± 3.34	15.86
Self-reported value	65.71 ± 11.69*	176.9 ± 7.16*	21.0 ± 3.15	14.05
Mean difference	−0.63 ± 3.91	−0.4 ± 2.94	−0.1 ± 2.10	
Age 15, girls (n = 620)				
Measured value	58.43 ± 9.98	166.7 ± 6.26	21.0 ± 3.13	11.45
Self-reported value	57.50 ± 9.71*	166.8 ± 6.19	20.6 ± 3.12*	8.87**
Mean difference	−0.93 ± 3.82	0.1 ± 2.11	−0.4 ± 1.47	

Mean difference was obtained by subtracting measured value from self-reported value

Overweight category includes obese adolescents, the IOTF cut-offs were used

* Significant difference from respective measured value; paired t-test, p < 0.001

** Significant difference from the prevalence of overweight based on measured data; test for one proportion, P < 0.05

The prevalence of overweight among 11–15-years-old schoolchildren based on self-reports was significantly lower compared to the prevalence using measured data in boys (16.4 versus 19.5 %, respectively; p = 0.026) and in girls (10.9 versus 14.9 % respectively; p < 0.001).

**Fig. 1:**
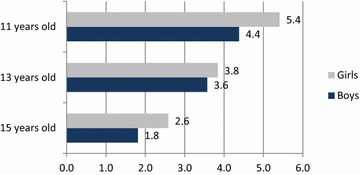
Bias in overweight prevalence (%) associated with self-reported weight and height by age and gender. During puberty years, the bias in overweight prevalence caused by self-reported weight and height decreases step by step, whereas the distinctive gender difference persists in all age groups

When age and gender were considered, overweight prevalence was underestimated by self-reports in all groups. The difference in percentage points was greatest among 11-year old girls and lowest among 15-year old boys. In all three age groups the differences were greater among girls. The differences between overweight status estimated using self-reported and measured weight and height values by age and gender are presented in Fig. 1.

Pearson correlation coefficients between self-reported and directly measured weight, height and BMI among boys were: 0.96, 0.95 and 0.88, respectively, and among girls 0.95, 0.95 and 0.89, respectively. By age and gender, the correlation coefficients were lowest in 11-year-old boys (0.94, 0.82 and 0.85, respectively) and girls (0.92, 0.90 and 0.85, respectively), and highest in 15-year-old boys (0.95, 0.91 and 0.90, respectively) and girls (0.93, 0.94 and 0.89, respectively). All correlations were strong and highly significant (p < 0.001).

**Fig. 2:**
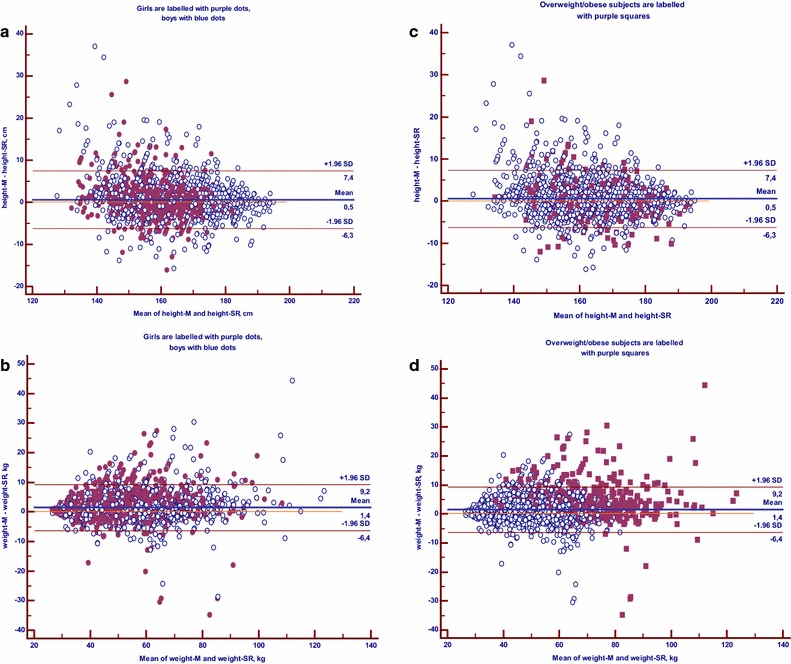
Bland–Altman plots of differences between measured and self-reported height and weight. **a**, **b** reflect Bland-Altman plots for height and weight, respectively, for boys and girls. **c**, **d** show also the plots for height and weight data, where points of overweight/obese subjects are highlighted with *purple square dots*. The *Blue dots* are the points of non-overweight subjects. The *continuous* *bold lines* in the graphs represent the mean differences, the *narrow lines* 95 % limits of agreement for whole sample (n = 3379), where upper limit is +1.96 SD and lower limit is −1.96 SD from the mean differences. *height-M and weight-M* directly measured height and weight, respectively, *height-SR and weight-SR* self-reported height and weight, respectively

To evaluate agreement between directly measured and self-reported anthropometric values in total study group (n = 3379) Bland–Altman plots were plotted. In Fig. 2, the differences between measured and self-reported height (panels a and c) or weight (Fig. 2b, d) values were plotted against the average of the measured and self-reported height or weight data, respectively. 95 % limits of agreement (LOA) between the two methods for the whole sample are shown in the figures. The distribution pattern of the height and weight data plot was similar for boys and girls (Fig. 2a, b). The LOA values (±1.96 SD) by gender, not shown in the figures, for height data were: −7.07 and 8.57 cm (boys); and: −5.41 and 6.10 cm (girls). The LOA values for weight were: −6.77 and 9.45 kg (boys); and: −5.98 and 9.02 kg (girls).

In the similar plot graphs for height and weight in panels c and d of Fig. 2 the data points of overweight subjects, estimated by BMI based on direct measurements of height and weight, are shown. Overweight subjects’ data points scatter homogeneously in the Bland–Altman plot for height (Fig. 2c). However, in the plot for weight, underestimation of weight status was observed for most of overweight participants (Fig. 2d). To characterize the level of agreement between measured and self-reported weight values among overweight subjects (n = 576) only the LOA for two methods were estimated for overweight participants (panels not shown). The LOA values for height in this group (−6.6 and 7.2 cm) were quite similar to the whole group (−6.3 and 7.4 cm, Fig. 2c). Agreement between measured and self-reported weight data among overweight subjects was different compared to the whole group: LOA values among overweight subjects was −8.9 and 16.8 kg (mean difference was 4 kg) while in the whole group the corresponding data were −6.4 and 9.2 kg (mean difference was 1.4 kg) (Fig. 2d).

The differences in prevalence of the three weight status classes based on directly measured versus self-reported measurements are presented in Table 2. In both genders overestimation of prevalence of underweight (for boys: by self-reports 2.3 % and by measured data 1.4 %, P = 0.0165; for girls: by self-reports 4.1 % and by measured data 2.2 %, P < 0.0001) and underestimation of overweight (for boys 16.4 % versus 19.5 %, respectively; p = 0.0018; for girls 10.9 % versus 14.9 %, respectively; p < 0.0001) were found when BMI was based on self-reported values.

**Table 2:** Classification of weight status classes estimated by measured and self- reported weight and height

Classification weight status based on measured data	Classification weight status based on self-reports				
	Underweight	Normal weight	Overweight^a^	n	%
Boys					
Underweight	12	10	0	22	1
Normal weight	24	1201	36	1261	79.1
Overweight	0	86	225	311	19.5
n	36	1297	261	1594	
%	2.3	81.3	16.4		
Girls					
Underweight	27	13	0	40	2.2
Normal weight	46	1408	26	1480	82.9
Overweight	73	97	168	265	14.9
n		1518	194	1785	
%	4.1	85.0	10.9		

^a^Overweight category includes also obese subjects

In the 3 × 3 contingency table (Table 2) including classifications of weight status categories based on directly measured and self-reported height and weight, the weighted Kappa statistics were calculated to assess the degree of agreement [30]. Kappa statistic for boys was 0.715 (95 % CI 0.67–0.76) and for girls 0.654 (95 % CI 0.61–0.70). By age and gender (data not shown) the weighted Kappa value was lowest among 13-year-old girls and highest among 15-year-old boys: 0.608 (95 % CI 0.52–0.70) and 0.721 (95 % CI 0.62–0.80), respectively.

Analysis of sensitivity and specificity of using self-reported data for estimation of overweight, showed that using BMI based on direct anthropometric measurements as the reference, the sensitivity of self-reported data for boys was 86.2 % and for girls 87.0 %, the specificity was 93.5 and 93.9 %, respectively. The positive predictive values for boys and girls were 72.3 and 63.4 %, respectively, and negative predictive value was 97.2 % for boys and 98.4 % for girls.

## Discussion

The current study showed an underestimation of weight based on self-reported data. It was associated with lower calculated BMI as well as overweight prevalence among adolescents, as has also been reported in other surveys [10–12, 16, 18, 21, 35]. The present survey also demonstrated under-reporting of height across all age and gender groups. The result is similar to Enes et al. [14], but differs to most other studies that show either over-reporting of height by adolescents [11, 15–17] or no consistent bias in height values [10, 18, 22, 36, 37]. In the present study, as well in previous studies, the bias of height through self-reporting is relatively small and unstable as compared to bias of weight values. Thus, despite instability in height biases in some studies the overweight prevalence based on self-reported BMI was underestimated in all of the above-cited studies. The Bland–Altman plots for weight and height in the whole sample and in overweight subjects only showed that most participants who were overweight based on actual measured weight status, under-reported their weight; and this bias was not noticeable for height.

The specific focus of the current study was to determine differences in the magnitude of bias in self-reported weight, height and BMI by age and gender during the adolescent years. A distinct age-related pattern in under-reporting of weight and height as well as estimated prevalence of overweight was found. The mean differences between self-reported and directly measured weight and height were greatest among 11-year-olds and lowest for those aged 15. Our results are consistent with a recent validation study where age-groups 11–13 and 14–17 years were compared and a greater underestimation of weight among younger children was found [18]. However, in a study of Chinese adolescents the opposite result was shown: an increasing underestimation of self-reported weight and BMI with increasing age [10]. One reason of this discrepancy may be due to differences in analytical methods applied. In terms of reporting bias in relation to gender, we found underestimation of weight and thus overweight prevalence in all age groups in both genders. The bias tended to be larger among girls as reported also in some previous studies [16, 18, 22, 37]. The causes of under-reporting weight by adolescents can be varied. Adolescents often report a desire to weigh less, as it is a social norm to be thin [18, 38], and this desire is greater among girls [38]. Besides the subjective cause there can also be some objective reasons for under-reporting of anthropometric values by adolescents, e.g. children can be unaware of their measurements. Students generally measure themselves infrequently and may only remember outdated values [14, 17, 39, 40]. This must be considered in particular for children during the period of pubertal growth spurt and weight gain [14, 26].

The difference between overweight prevalence based on self-reported versus directly measured BMI varied among European countries from −2.3 to −15 %; these studies used varying protocols to measure weight [18, 23]. In the study with the lowest bias [18] the direct measurements were taken in underwear and self-reported weight and height were asked face-to-face prior to the direct measurements. However, in the study with the largest difference between overweight prevalence based on self-reported and directly measured BMI [23], the weighing procedure involved subtracting 1 kg from each child’s weight to account for clothes worn (age was not considered). Also, the direct measurements were taken 3 months later than self-reported data. Thus, another source of bias in BMI and overweight prevalence in validation studies is study protocols. Differences in when and how direct measurements (in light clothing versus underwear only) are taken and whether corrections for cloths are used can impact on validation with self-report measures [18, 23].

It was considered that accuracy of reporting height and weight may be higher when students are aware that direct measurements will be taken afterwards [8, 14]. However, in a recent study in adults this hypothesis was not confirmed [41]. This needs to be examined further for adolescents in future investigations.

In the current study, Bland–Altman plots demonstrated minimal mean differences between the two methods (direct versus self-reported measurements) for height (0.5 cm). The mean difference between the methods for weight was notable (1.5 kg), suggesting a systematic under-reporting of weight.

Classifications of underweight, normal weight and overweight, based on measured and self-reported BMI have shown good agreement based on the Kappa statistics. In the current study, sensitivity, i.e. the proportion of actual overweight (includes obese) adolescents who were classified correctly using self-reported BMI, was 86.6 % (86.2 % for boys and 87 % for girls). This is higher than shown in previous validation studies, where the sensitivity values varied between 52.2 and 74.8 % [10, 14, 18, 21, 36]. In the present study specificity was 93.7 %, demonstrating that a certain number (~6 %) of non-overweight students were incorrectly classified as overweight using BMI based on self-reported data. Direct comparison of sensitivity and specificity values with other studies is not feasible because national cut-off points for the BMI categories were often used [14, 15, 18] and/or the values were presented for overweight and obese subjects separately [14, 15, 21, 36]. In previous studies positive predictive value, indicating the likelihood of diagnosing overweight accurately by self-reports, was rarely reported [10, 36]. In the present study the positive predictive value was 72 % for boys and 63 % for girls. This finding is in accordance with the observed greater bias of overweight prevalence among girls in all age groups.

This study has certain strengths and limitations. An advantage of the current survey is using a representative study sample, in which it was possible to study age and gender differences in biases of self-reported anthropometric measures through the period of puberty. The strength is also that great attention was given to avoid potential bias that can be introduced by inaccuracies in procedures of taking measurements: there was no lapse of time between collecting self-reported height and weight and carrying out direct measurements; identical new equipment was used throughout the country, and a standard protocol was used to consider weight of clothes worn during direct measurements.

In this study students were aware that their height and weight would be subsequently measured, which is considered to lead to more accurate reporting than in usual HBSC study. However, the supposition was not confirmed in a study among adults [41].

## Conclusions

In the current study, certain age-related patterns in under-reporting of weight and height values as well as estimated overweight prevalence were found across the period of adolescence. The mean differences between self-reported and directly measured weight, height and BMI were greatest among 11-year-olds and lowest for those aged 15 years. The underestimation of overweight prevalence showed a linear trend to decrease with increasing age. The extent of misclassification in height and its impact on the bias of overweight prevalence were less than that of weight. The mean underestimation of overweight prevalence based on self-reports was rather small, 3.6 %. BMI basing on self-reported data remains the method of choice in large surveys for practical and logistical reasons.

## Additional file

10.1186/s13104-015-1587-9 Rules of weighting.

## Abbreviations

BMI: body mass index
HBSC: Health Behaviour of School-aged children
CINDI: Countrywide Integrated Noncommunicable Diseases Intervention
height-M, weight-M, BMI-M: directly measured height, weight and resulting BMI
height-SR, weight-SR, BMI-SR: self-reported height, weight and resulting BMI
LOA: limits of agreement
CI: confidence interval
