# Supplementary material for: Validity of self-reported height and weight for estimating prevalence of overweight among Estonian adolescents: the Health Behaviour in School-aged Children study
Source: BMC Res Notes. 2015 Oct 26;8:606. doi: 10.1186/s13104-015-1587-9 (PMC4621857; doi:10.1186/s13104-015-1587-9)
Supplement: Supplementary file 1 — 10.1186/s13104-015-1587-9 Rules of weighting. [file 13104_2015_1587_MOESM1_ESM.pdf]

Instruction.

Rules for calculation weight of clothes used in Estonian HBSC survey

The pupils were asked to take off thick jumpers, cardigans and jackets.

Approximate weight of clothes:

Jeans (mobile phone, purse - had to be taken out of pockets; a thick belt with a big buckle was calculated to weigh 50-100 g):

- of a smaller child - 400-500g
- of a bigger child - 600g (thin material) - 800g (thick material)
- of a tall boy, of thick material having a thick belt with a big buckle - 900-1000g

Tight trousers or leggings of thin material:

- of a smaller child - 300g
- of a bigger child - 400g

Warmer jumper, jacket:

- of a smaller child - 300g
- of a bigger child - 400-500g

Shirt, blouse with long sleeves: - 200-250g

Jumper, knitted garment of thinner material:

- of a smaller child - 200g
- of a bigger child - 300-350g

T-shirt

- of a smaller child - 150g
- of a bigger child - 200-250g
